# Genome-Wide Characterization of Calmodulin-Binding Transcription Activators Genes in *Aegilops tauschii*

**DOI:** 10.3390/ijms27177790

**Published:** 2026-08-31

**Authors:** Elnaz Nozari, Rasool Asghari-Zakaria, Nasser Zare, Parisa Sheikhzadeh, Mehran Nozari-Asbemarz

**Affiliations:** 1 Agricultural Biotechnology Program, Department of Plant Production and Genetics, Faculty of Agriculture and Natural Resources, University of Mohaghegh Ardabili, Ardabil 56199-11367, Iran; 2 Department of Chemical Sciences, Bernal Institute, University of Limerick, V94 T9PX Limerick, Ireland

**Keywords:** CAMTA, transcription factors, water-deficit stress, cis-regulatory elements, qRT-PCR

## Abstract

Calcium signaling plays a central role in plant adaptation to abiotic stresses and is primarily mediated by calmodulin and its associated transcription factors. Calmodulin-binding transcription activators (CAMTAs) regulate stress-responsive gene expression, but their characteristics and functions remain largely unexplored in *Aegilops tauschii* Coss., the D-genome progenitor of bread wheat. In this study, a genome-wide identification and characterization of the *CAMTA* gene family was performed, followed by phylogenetic, structural, conserved domain, promoter cis-element, and expression analyses under drought stress. Five *AetCAMTA* genes were identified and classified into three phylogenetic groups. All proteins contained conserved CG-1 DNA-binding, ankyrin repeat (ANK), and IQ calmodulin-binding domains and exhibited similar exon–intron organization. Promoter analysis revealed abundant hormone- and stress-responsive cis-elements, particularly abscisic acid-responsive element (ABRE) and drought-responsive MYB-binding site (MBS) motifs, suggesting their involvement in drought-responsive signaling. Quantitative RT-PCR showed genotype- and stress-dependent expression patterns, with the drought-tolerant ecotype (TN-01-1747) exhibiting higher expression of *AetCAMTA1*, *AetCAMTA2*, and *AetCAMTA3* than the drought-sensitive ecotype (TN-01-1559) under moderate drought stress. These findings provide new insights into the evolutionary and functional characteristics of *AetCAMTA* genes and identify promising candidates for improving drought tolerance in wheat through molecular breeding and biotechnological approaches.

## 1. Introduction

Abiotic and biotic stresses are primary limiting factors for crop productivity worldwide, with drought representing one of the most severe constraints on agricultural systems. Water deficit negatively affects plant growth by disrupting photosynthesis, metabolic activity, and cellular homeostasis, ultimately leading to significant reductions in biomass and yield. Plant responses to drought are highly dynamic and depend on genotype, developmental stage, and stress intensity [1,2]. A key early signaling event in plant responses to environmental stimuli is the transient increase in cytosolic calcium ion (Ca^2+^) concentration. These Ca^2+^ fluctuations, known as “calcium signatures”, encode information that enables plants to perceive and respond specifically to diverse stresses [3,4,5,6]. In plants, Ca^2+^ functions as a pivotal intracellular second messenger, orchestrating a wide range of developmental processes and adaptive responses to environmental stimuli [7]. Calmodulin (CaM) is one of the most essential Ca^2+^ sensor proteins and serves as a central component in decoding these signals. Upon elevation of intracellular Ca^2+^ levels, CaM undergoes conformational changes and interacts with a wide range of target proteins, including transcription factors, thereby regulating the expression of genes involved in growth, development, and stress adaptation [8,9,10,11,12].

More than 90 CaM-binding transcription factors have been identified in plants, among which CAMTAs represent a highly conserved and functionally vital family [12,13]. CAMTA proteins are characterized by multiple conserved domains, including the CG-1 DNA-binding domain, the Transcription-associated Immunoglobulin-like (TIG) domain, ANK, and calmodulin-binding IQ and Ca^2+^-dependent CaM-binding (CaMBD) domains [12,14,15]. The CG-1 domain enables sequence-specific binding to cis-elements such as (A/C)CGCG(C/G/T), allowing *CAMTAs* to directly regulate the transcription of downstream stress-responsive genes [16]. Although most CAMTAs possess a conserved domain architecture, variations in domain composition—such as partial or missing domains—have been reported, which may contribute to functional diversification under varying environmental conditions [6].

Genome-wide analyses have identified the CAMTA gene family in numerous plant species, including *Arabidopsis thaliana*, *Oryza sativa*, *Brachypodium distachyon*, *Glycine max*, *Brassica napus*, *Citrus* spp., *Musa acuminata*, *Camellia sinensis*, and *Triticum aestivum*, demonstrating that CAMTA genes are evolutionarily conserved across diverse plant lineages [1,6,8,15,17,18,19,20,21]. Nevertheless, lineage-specific expansion has resulted in considerable variation in CAMTA family size among plant species. Functional studies have demonstrated diverse roles of CAMTA genes in plant stress responses and immunity. In wheat, several *TaCAMTA* genes are differentially expressed under drought stress, suggesting their involvement in drought-response pathways [15]. In tea plant, *CsCAMTA1–3* responds to cold, salinity, and Polyethylene glycol (PEG)-induced drought, while *CsCAMTA2* is additionally induced by methyl jasmonate (MeJA), indicating roles in both abiotic and biotic stress signaling [21]. In *Arabidopsis*, CAMTAs act as key regulators of plant immunity by modulating the biosynthesis of pipecolic acid (Pip), salicylic acid (SA), and N-hydroxypipecolic acid (NHP) biosynthesis pathways and regulating defense-related genes such as *ALD1*, *SARD1*, and *CBP60g* [22,23,24,25]. CAMTA proteins have also been implicated in salt tolerance, cold acclimation, freezing resistance, drought adaptation, herbivore defense, and aluminum tolerance through the regulation of CBF-, auxin-, JA-, and SA-associated signaling pathways [26,27,28,29,30,31,32,33]. Moreover, *GmCAMTA12* enhances drought tolerance in soybean [34], *TaCAMTA4* contributes to resistance against *Puccinia triticina* in wheat [35], and *BnCAMTA3A1* and *BnCAMTA3C1* are induced during infection by *Sclerotinia sclerotiorum* in rapeseed [19]. CAMTAs constitute part of the complex regulatory networks that govern plant responses to environmental stresses like drought, salinity, and extreme temperatures, as well as biotic stresses caused by pathogens and herbivores. These stresses negatively impact plant growth and crop yield [36,37]. Plant adaptation involves the coordinated regulation of various transcription factor families and stress-responsive genes. Besides CAMTAs, other transcription factors, such as NACs, MYBs, and WRKYs, are crucial for abiotic stress tolerance. For example, overexpression of *FvNAC29* from *Fragaria vesca* enhanced salt and cold tolerance in *Arabidopsis thaliana*, accompanied by changes in antioxidant activity and the expression of stress-responsive genes [38]. Similarly, *MbWRKY3* from *Malus baccata* is induced by drought, salinity, and abscisic acid (ABA), and its overexpression enhances drought tolerance in transgenic tobacco [39]. More recently, *MbWRKY50* was shown to promote cold and drought tolerance through enhanced antioxidant capacity and the activation of abscisic acid (ABA)- and stress-responsive pathways [40]. In addition to transcriptional regulators, functional genes involved in nutrient homeostasis can also contribute to abiotic stress adaptation. Overexpression of *MxFRO6* from *Malus xiaojinensis* increased iron and salt tolerance in *Arabidopsis thaliana*, whereas overexpression of *MxCS1* enhanced iron-stress tolerance in tobacco, highlighting the contribution of diverse molecular mechanisms to plant stress adaptation [41,42].

Bread wheat (*Triticum aestivum* L.) is a major staple crop that provides food for more than 4.5 billion people worldwide [43]. Ensuring global food security under conditions of climate change, population growth, and declining genetic diversity requires the effective utilization of diverse genomic resources [44]. Wild relatives of wheat represent valuable reservoirs of genetic variation for crop improvement. Among them, *Ae. tauschii*, a diploid species (2*n* = 2*x* = 14, DD genome), is particularly important because it contributed the D genome to modern bread wheat. This species harbors a wide range of agronomically important traits, including resistance to multiple diseases and tolerance to environmental stresses [45]. Several resistance genes, such as *Sr33*, *Yr28*, and *Pm35*, have already been introgressed from *Ae. tauschii* into cultivated wheat [46,47]. However, its potential as a genetic source for improving drought tolerance remains insufficiently explored. Previous studies have reported that *Ae. tauschii* and other *Aegilops* species exhibit notable drought tolerance [48,49], highlighting their potential utility for enhancing abiotic stress resilience in wheat breeding programs.

Drought stress severely affects plant growth and productivity by disrupting photosynthesis, metabolism, and oxidative balance [50,51,52]. Therefore, drought tolerance indices have been widely used to identify stress-resilient genotypes based on plant performance under both normal and stress conditions [53]. Several indices, including tolerance index (TOL), mean productivity (MP), stress susceptibility index (SSI), geometric mean productivity (GMP), and stress tolerance index (STI), have been proposed for evaluating drought tolerance [54,55,56]. Among these, STI is considered one of the most effective criteria for identifying genotypes that maintain high yield under both optimal and drought conditions. Therefore, the present study aimed to comprehensively identify and characterize the CAMTA gene family in *Ae. tauschii*, including their genomic distribution, conserved motifs, gene structure, and cis-regulatory elements, and to explore the potential involvement of selected *AetCAMTA* genes in drought response. In addition, the expression profiles of selected *AetCAMTA* genes were investigated in contrasting drought-tolerant and drought-sensitive genotypes, which were identified based on plant dry weight (PDW) and STI under drought stress conditions.

## 2. Results

### 2.1. Plant Growth

**Figure 1:**
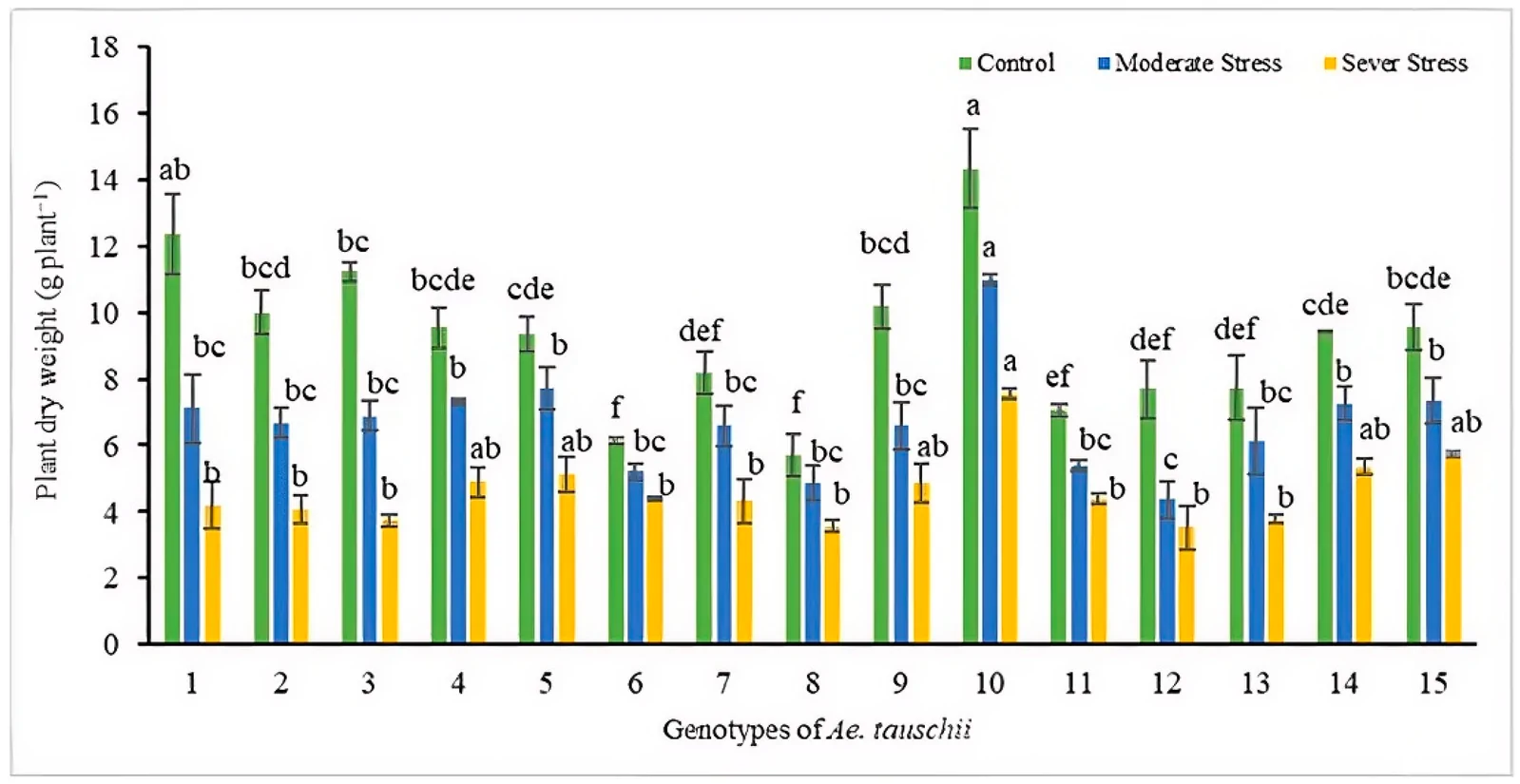
PDW (g plant^−1^) in 15 *Ae. tauschii* ecotypes under well-watered, moderate, and severe water stress treatments. Means with nonsimilar letters show significant differences at a 1% probability level. Error bars show standard errors (*n* = 3).

Analysis of variance revealed that irrigation regime, ecotype, and their interaction significantly affected PDW in *Ae. tauschii* (*p* < 0.01), indicating significant genetic variation in drought response among the evaluated ecotypes (Table 1). Across all genotypes, PDW progressively decreased with increasing drought severity. However, the magnitude of reduction varied considerably among ecotypes, reflecting differences in drought sensitivity and growth stability under water-limited conditions. Among the evaluated genotypes, ecotype 10 maintained the highest PDW under both moderate (10.99 g) and severe drought stress (7.57 g), suggesting greater biomass stability under contrasting moisture conditions (Figure 1). These findings demonstrated considerable phenotypic diversity in drought adaptation among *Ae. tauschii* ecotypes and provided the basis for subsequent drought tolerance index analyses. 

### 2.2. Evaluation of Drought Stress Tolerance

**Figure 2:**
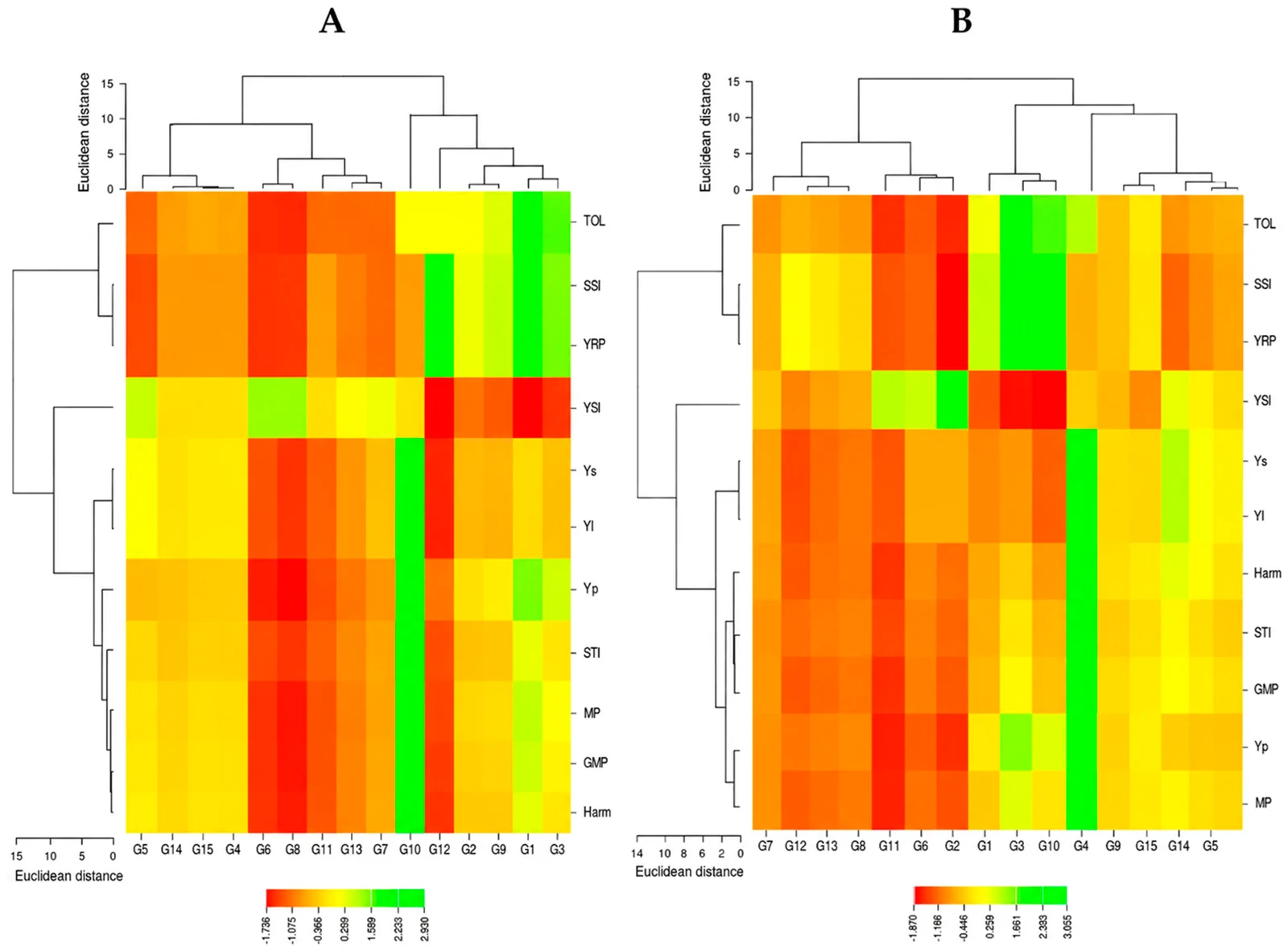
Clustering and heat map of 15 *Ae. tauschii* ecotypes under (**A**) moderate water stress and (**B**) severe water stress conditions.

Drought tolerance indices calculated from PDW under stress and non-stress conditions revealed substantial variation in drought tolerance indices among the 15 *Ae. tauschii* ecotypes. Hierarchical clustering and heatmap analyses separated the ecotypes into distinct groups according to their productivity and stress responsiveness (Figure 2A,B). Under moderate drought stress, ecotype 10 formed a distinct cluster characterized by the highest values of productivity- and stability-related indices, including YS, YI, YP, STI, MP, GMP, and HARM, together with relatively low stress susceptibility values. This pattern confirmed its superior drought tolerance and stable performance under water-deficit conditions. In contrast, ecotypes 1, 2, 3, 7, 8, 11, 12, and 13 exhibited comparatively lower productivity-related index values and higher susceptibility indices, indicating greater sensitivity to moderate drought stress. The remaining ecotypes displayed intermediate responses, suggesting moderate tolerance and partial yield stability (Figure 2A).

Similar clustering pattern was observed under severe drought stress conditions. Ecotype 10 consistently maintained the highest productivity and stability indices, confirming its superior adaptation to water-limited environments. Conversely, ecotypes 6, 7, 8, 11, 12, and 13 exhibited the lowest performance across most drought tolerance indices and were classified as highly sensitive genotypes. Ecotypes 4, 5, 9, 14, and 15 displayed intermediate responses under severe stress conditions, indicating moderate drought tolerance (Figure 2B). Among the evaluated indices, STI effectively discriminated drought-tolerant and drought-sensitive genotypes. Based on STI values, ecotype TN-01-1747 exhibited the highest drought tolerance, whereas TN-01-1559 showed the lowest performance under stress conditions, highlighting the reliability of STI for identifying stable and high-performing genotypes across contrasting moisture regimes.

### 2.3. Identification and Physicochemical Characterization of AetCAMTA Genes

A total of five CAMTA genes were identified in the *Ae. tauschii* genome and designated as *AetCAMTA1* to *AetCAMTA5* according to their sequence similarity with previously characterized CAMTA genes in rice. Chromosomal mapping revealed that the identified genes were distributed across three chromosomes. *AetCAMTA3*, *AetCAMTA4*, and *AetCAMTA5* were located on chromosome 2D, whereas *AetCAMTA1* and *AetCAMTA2* were mapped to chromosomes 3D and 4D, respectively. The physicochemical properties of the predicted *AetCAMTA* proteins are summarized in Table 2. Protein length ranged from 832 amino acids in *AetCAMTA1* to 1066 amino acids in *AetCAMTA2*, with predicted molecular weights varying between 94.18 and 119.16 kDa. Exon numbers ranged from 11 to 13, with *AetCAMTA2*, *AetCAMTA3*, and *AetCAMTA5* each containing 13 exons.

Most *AetCAMTA* proteins exhibited acidic isoelectric points (pI < 7), whereas *AetCAMTA5* displayed a basic (pI = 9.00) value. Instability index analysis predicted that all proteins except *AetCAMTA1* and *AetCAMTA5* were structurally unstable as their instability indexes exceeded 40. In addition, all identified proteins exhibited negative GRAVY values, indicating hydrophilic characteristics. The extinction coefficients of the *AetCAMTA* proteins ranged from 121,530 M^−1^ cm^−1^ for *AetCAMTA1* to 148,545 M^−1^ cm^−1^ for *AetCAMTA2*. According to an in silico study, subcellular localization analysis further predicted that all *AetCAMTA* proteins are localized in the nucleus, consistent with their predicted function.

### 2.4. Phylogenetic Analysis of AetCAMTA Proteins

**Figure 3:**
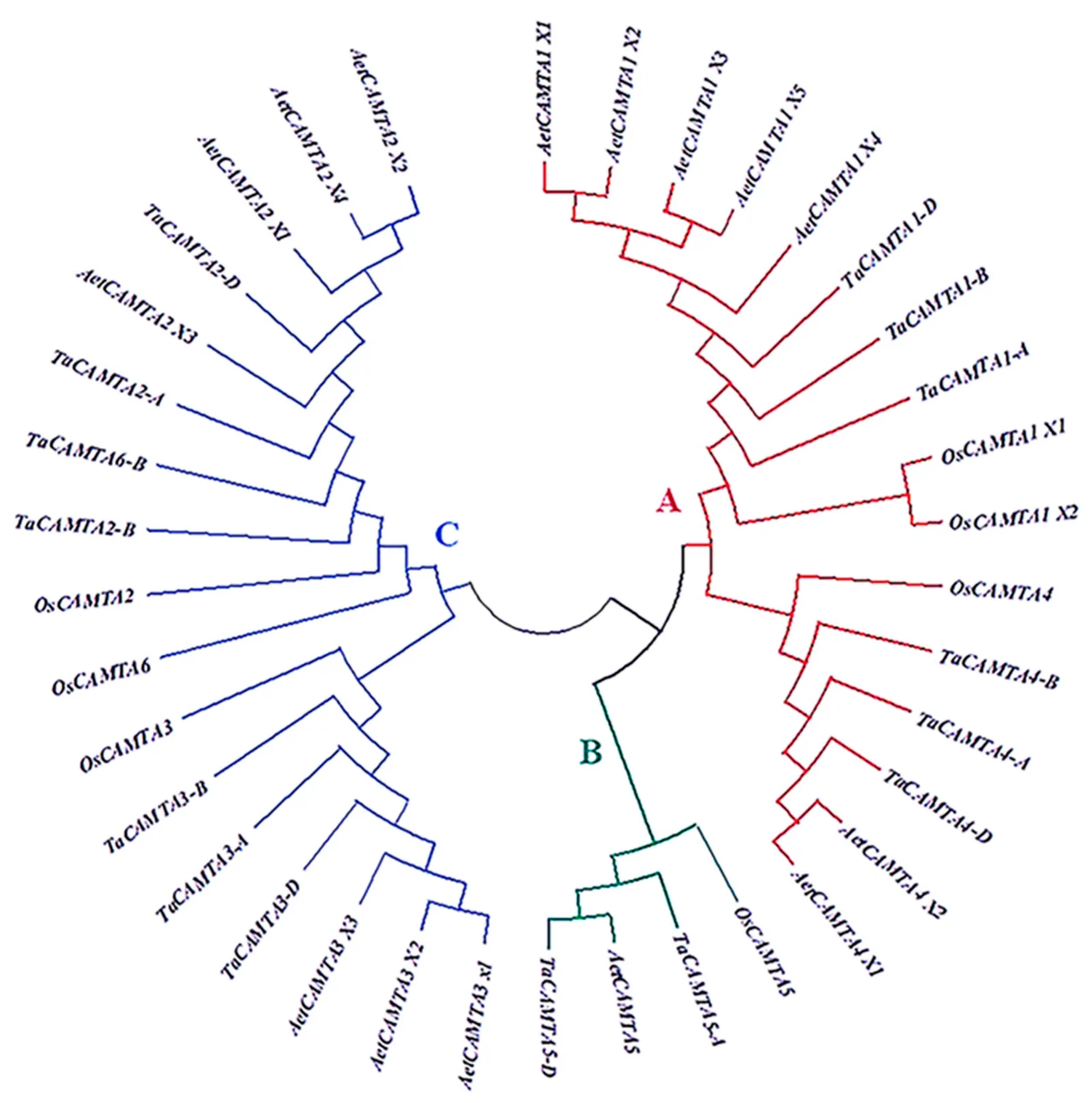
Phylogenetic relationships of *CAMTA* proteins from *Ae. tauschii*, *Triticum aestivum*, and *Oryza sativa* subsp. japonica. The proteins were classified into three major groups (A–C). Group A contains CAMTA1 and CAMTA4 homologs, Group B contains CAMTA5 homologs, and Group C contains CAMTA2 and CAMTA3 homologs from the three species.

To investigate the evolutionary relationships of the *CAMTA* gene family, a phylogenetic tree was constructed using full-length CAMTA protein sequences from *Ae. tauschii*, *Triticum aestivum*, and *Oryza sativa japonica*. The phylogenetic analysis classified all CAMTA proteins into three major groups designated as Groups A, B, and C (Figure 3). Group A contained *AetCAMTA1* and *AetCAMTA4* together with their corresponding wheat homologs (*TaCAMTA1* and *TaCAMTA4*) and rice homologs (*OsCAMTA1* and *OsCAMTA4*). Group B included *AetCAMTA5* clustered with *TaCAMTA5* and *OsCAMTA5*. Group C comprised *AetCAMTA2* and *AetCAMTA3* together with *TaCAMTA2*, *TaCAMTA3*, *OsCAMTA2, OsCAMTA3*, and *OsCAMTA6*. The close clustering of *AetCAMTA* proteins with both wheat and rice homologs indicated strong evolutionary conservation of the CAMTA family across grass species.

### 2.5. Gene Structure Analysis

**Figure 4:**
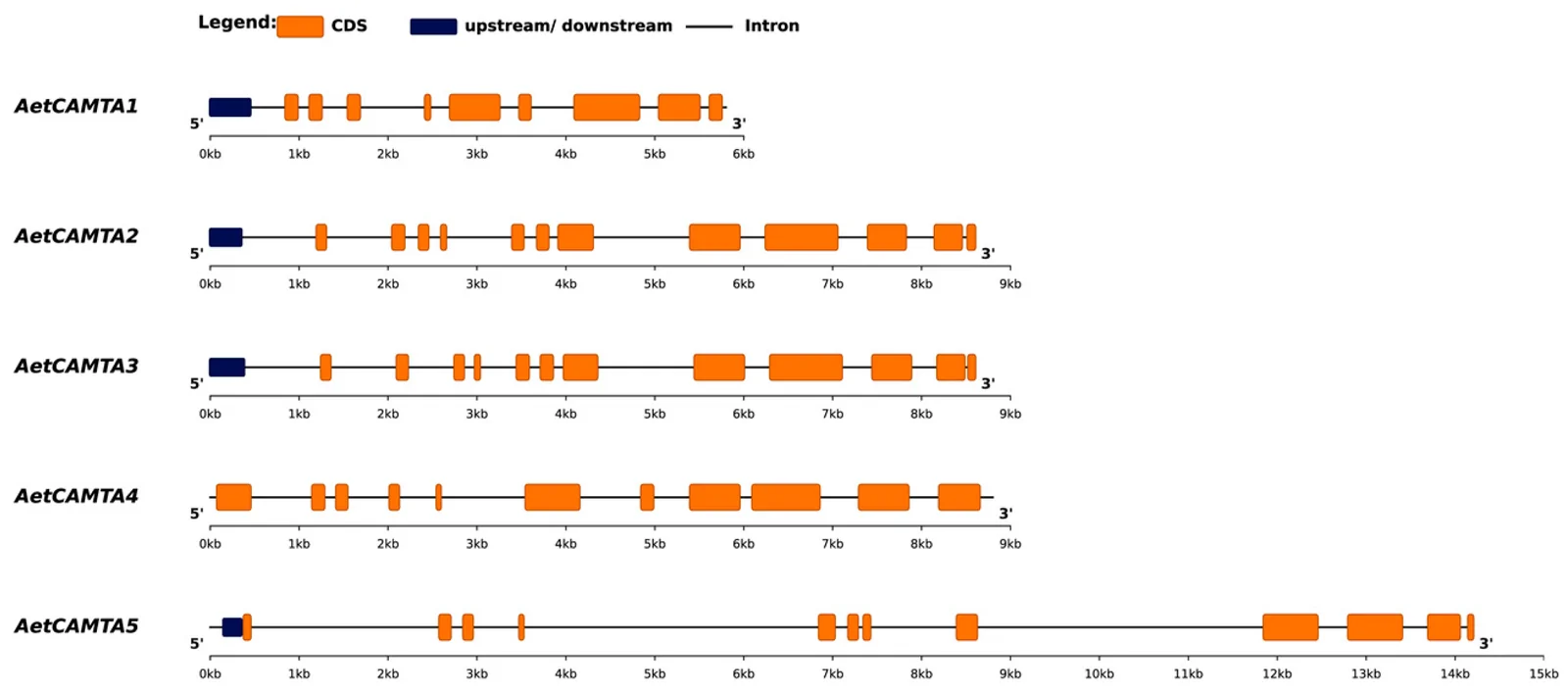
Exon–intron structural organization of the AetCAMTA gene family (*AetCAMTA1* to *AetCAMTA5*). Schematic representation illustrating the genomic architecture of five AetCAMTA genes plotted along their physical genomic length in kilobases (kb). Coding sequences (CDS/exons) are represented by golden-yellow rounded boxes, untranslated regions (5′/3′ UTR; upstream/downstream regulatory regions) are designated as dark blue rectangles, and intervening introns are indicated by solid black horizontal lines. The relative 5′ to 3′ transcriptional orientation is annotated for each locus, with aligned scale bars (in kb) positioned directly below each gene model.

Gene structure analysis further revealed a relatively conserved exon–intron organization among *AetCAMTA* genes (Figure 4). The number of introns ranged from 10 to 12 across the five identified genes. Exons, introns, and untranslated regions (5′ and 3′ UTRs) displayed similar structural arrangements, indicating a conserved gene organization within the *AetCAMTA* gene family.

### 2.6. Conserved Motif and Domain Architecture Analysis of AetCAMTA Proteins

**Figure 5:**
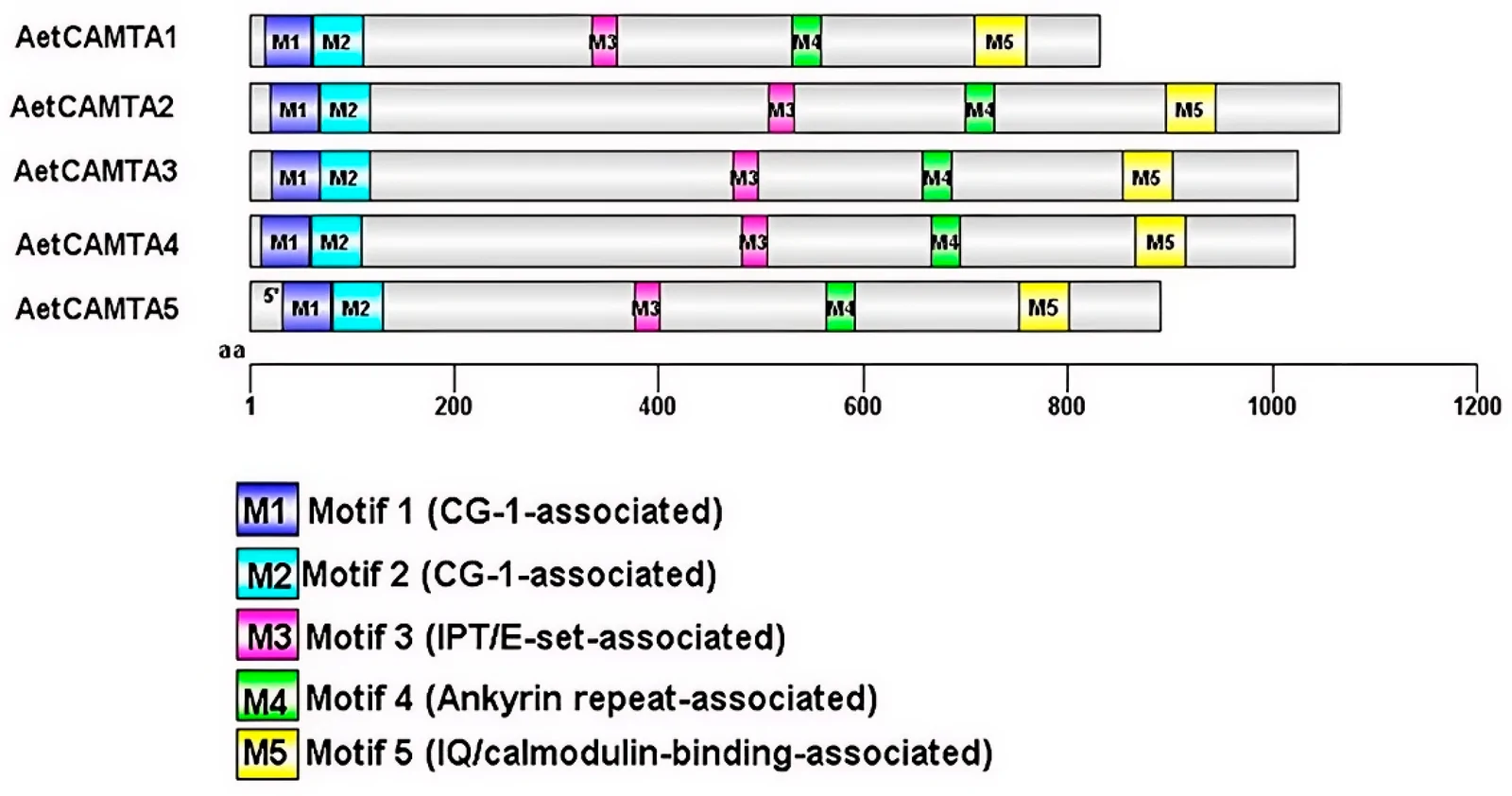
Schematic distribution of conserved protein motifs in *Aegilops tauschii* CAMTA transcription factors (*AetCAMTA1*–*AetCAMTA5*). Conserved structural motifs were predicted using the MEME suite and mapped along the full-length deduced amino acid (aa) sequences. Individual proteins are represented by grey rounded backbones, with lengths indicated by the bottom amino acid scale bar (ranging from 1 to 1200 aa). Conserved motifs are highlighted as numbered, color-coded blocks.

**Figure 6:**
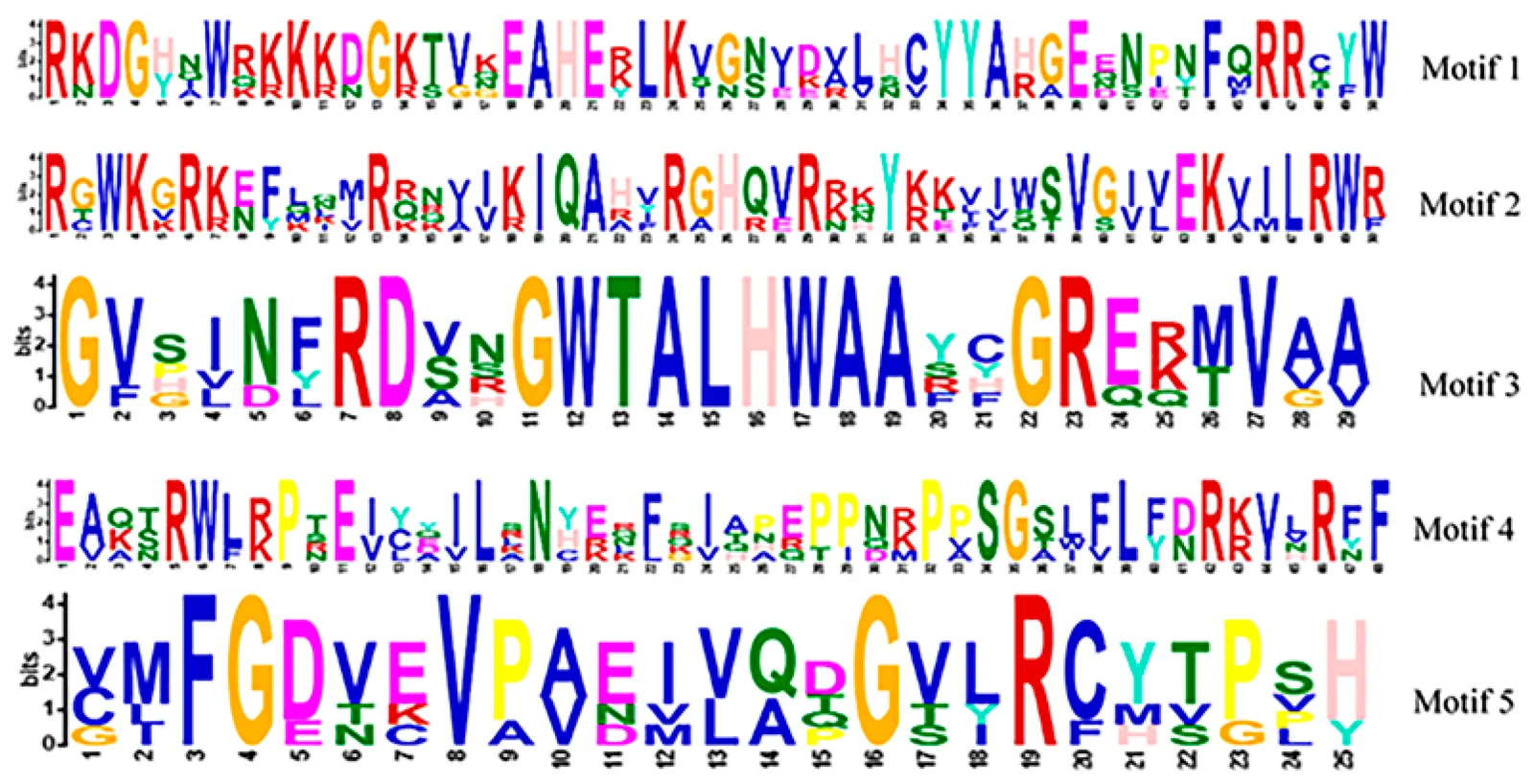
Sequence logo analysis of conserved amino acid motifs identified in *Aegilops tauschii* CAMTA (AetCAMTA) transcription factor proteins. The conserved motifs (Motifs 1 to 5) were identified and characterized using the MEME suite. The horizontal axis indicates the residue position within each motif, while the total height of each stack on the vertical axis represents sequence conservation measured in bits (information content, ranging from 0 to ≈4.3 for amino acids). The relative height of each individual single-letter amino acid code within a stack corresponds to its relative frequency at that specific position.

Conserved motif analysis identified five highly conserved motifs (M1–M5) across all AetCAMTA proteins (Figure 5 and Figure 6). The detected motifs showed extremely significant E-values ranging from 6.7 × 10^−905^ to 1.8 × 10^−29^, indicating strong sequence conservation among members of the CAMTA family. Functional annotation revealed that Motifs 1 and 2 corresponded to the CG-1 DNA-binding region, Motif 3 was associated with the TIG/IPT-related domain, Motif 4 represented ANK sequences, and Motif 5 corresponded to the IQ calmodulin-binding motif.

Motif distribution analysis revealed a largely conserved structural organization within the AetCAMTA protein family. Motifs 1 and 2 were consistently located in the N-terminal region, whereas Motifs 3–5 occupied relatively conserved central and C-terminal regions. Despite minor variation in motif positions, all AetCAMTA proteins retained the complete motif set, with no apparent motif gain or loss.

**Figure 7:**
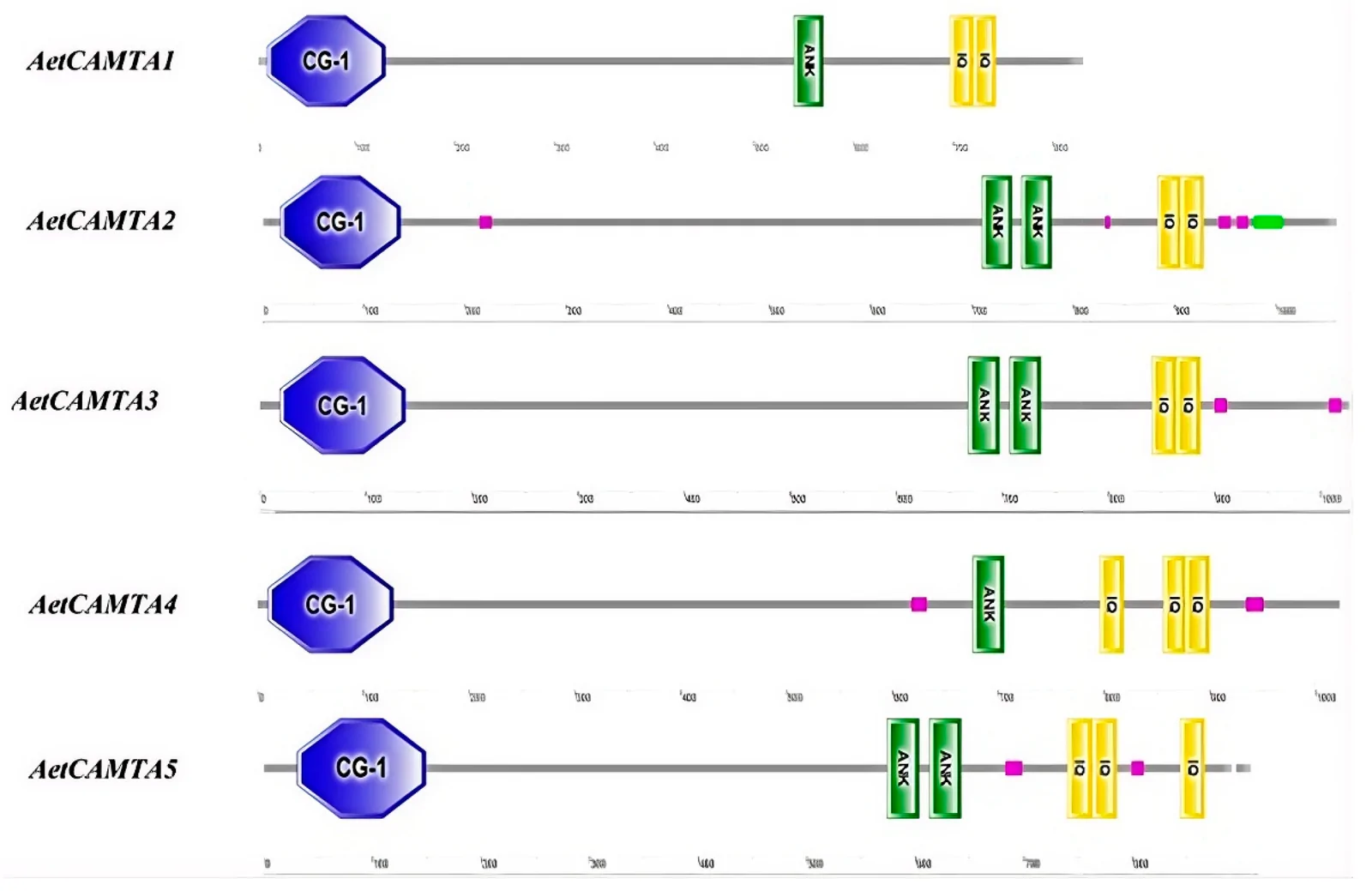
Protein domain composition and structural organization of CAMTA transcription factors in *Aegilops tauschii* (*AetCAMTA1*–*AetCAMTA5*). Schematic representation illustrating the characteristic functional domains along the deduced amino acid sequences of five AetCAMTA proteins. Protein backbones are represented as grey horizontal lines, with relative lengths and domain positions mapped against the bottom amino acid (aa) scale bar (0–1000 aa).

Domain analysis further confirmed that all AetCAMTA proteins possess the characteristic CAMTA domain architecture, including the CG-1 DNA-binding domain, ANK domains, and IQ calmodulin-binding motifs organized along the protein sequence. All proteins contained a conserved CG-1 domain of approximately 130 amino acids together with a bipartite nuclear localization signal (NLS). Variation was observed in the number of ANK and IQ motifs among family members. *AetCAMTA1* and *AetCAMTA4* contained a single ANK region, whereas *AetCAMTA2*, *AetCAMTA2*, and *AetCAMTA5* possessed two ANK domains. In addition, *AetCAMTA4* and *AetCAMTA5* contained three IQ motifs, whereas *AetCAMTA1*–*AetCAMTA2* contained two IQ motifs (Figure 7). Detailed motif composition, motif coordinates, and conserved domain annotation for each AetCAMTA protein are provided in Supplementary Table S1, while motif annotation and corresponding E-values are presented in Supplementary Table S2.

Notably, motif discovery and domain annotation were not completely identical. While MEME identified conserved sequence motifs across the proteins, not all detected motifs were retained as annotated domains in SMART analysis. This difference likely reflects methodological distinctions, as MEME detects statistically conserved sequence patterns independently of predefined domain models, whereas SMART applies profile-based criteria for domain assignment. Consequently, only SMART-supported domains were used for domain mapping and structural visualization. 

### 2.7. Secondary and 3D Structure Analysis of AetCAMTA Proteins

**Figure 8:**
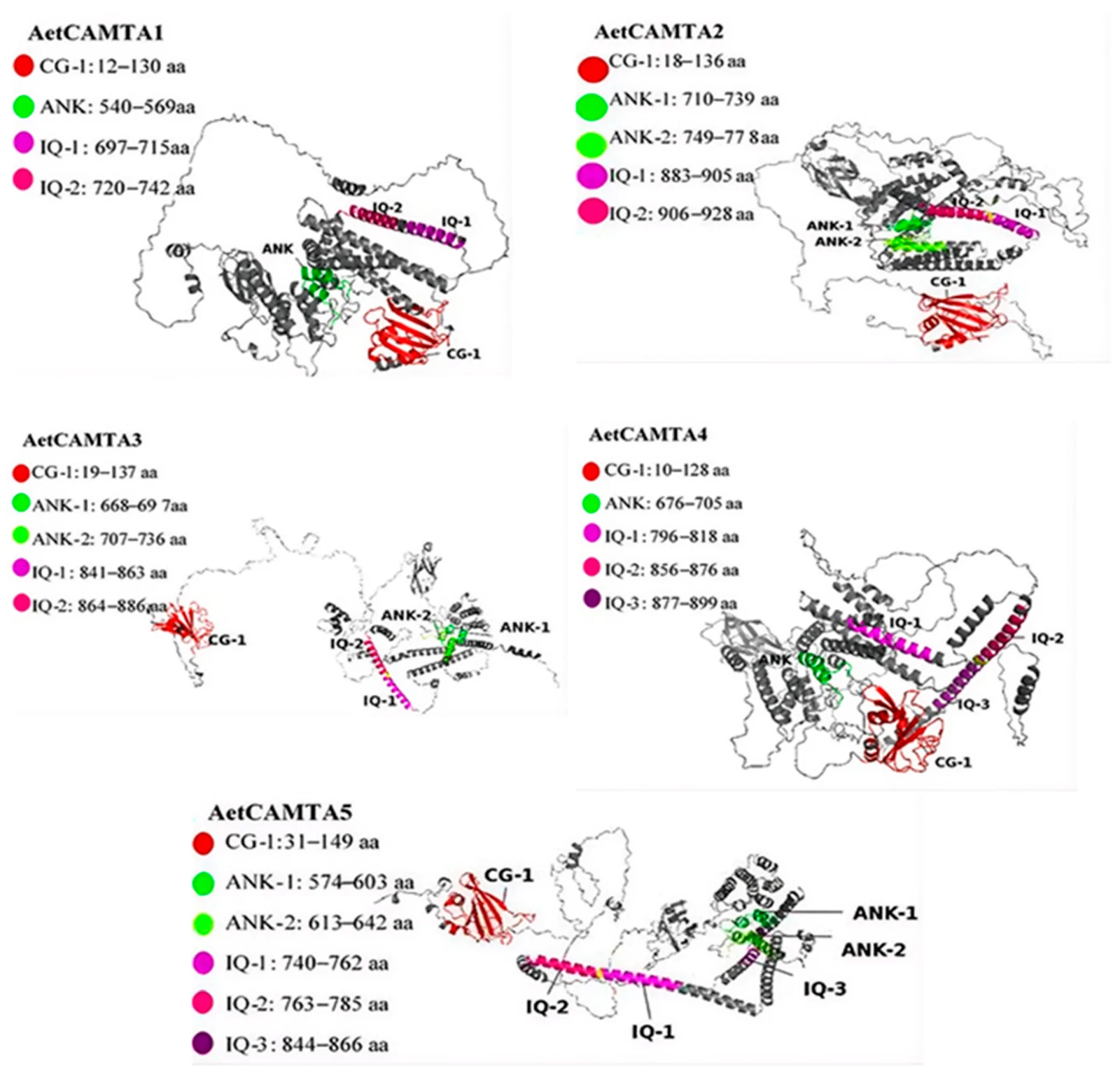
Predicted three-dimensional (3D) tertiary structures and domain mapping of CAMTA transcription factors in *Aegilops tauschii* (*AetCAMTA1*–*AetCAMTA5*). Structural backbones and disordered/loop regions are shown in grey ribbon/cartoon representations. Distinct functional domains are color-coded with exact amino acid (aa) positional coordinates specified for each protein.

Secondary structure prediction revealed that all AetCAMTA proteins shared a similar structural composition predominantly consisting of α-helices and coil regions, whereas β-strands represented a comparatively smaller proportion of the total structure (Table 3). Among the identified proteins, *AetCAMTA5* exhibited the highest α-helical content (42.98%), whereas *AetCAMTA2* displayed the lowest proportion of α-helices (34.21%). Coil regions represented the predominant structural component across all proteins, ranging from 49.63% to 59.75%, indicating the presence of flexible regions potentially involved in regulatory interactions. In contrast, β-strands accounted for only 4.59–9.13% of the predicted secondary structure. To further investigate structural features, full-length AetCAMTA proteins were subjected to three-dimensional structure modeling followed by structural refinement and energy minimization (Figure 8). The predicted models showed similar global folds characterized predominantly by α-helical assemblies interconnected through loop-rich regions, consistent with the secondary structure predictions.

### 2.8. Cis-Acting Elements in the Promoter Regions of AetCAMTA Genes

Promoter analysis of *AetCAMTA* genes identified 35 types of cis-acting regulatory elements associated with hormone signaling, stress responsiveness, and light-mediated regulation. A 1500-bp upstream region from the translation start site of each gene was analyzed using the PlantCARE database. Between 1 and 75 cis-elements were detected across the promoter regions of the five *AetCAMTA* genes (Table 4). Hormone-responsive elements included abscisic acid-responsive elements (ABRE, ABRE3a, and ABRE4), auxin-responsive elements (AuxRR-core and TGA-element), methyl jasmonate-responsive motifs (CGTCA-motif), and gibberellin-responsive elements (GARE-motif and P-box). Among these, ABRE motifs were the most abundant hormone-related cis-elements detected in *AetCAMTA* promoters. Several stress-responsive elements were also identified, including anaerobic response elements (ARE and GC-motif), low-temperature-responsive elements (LTR), MBS, and pathogen- or wound-responsive motifs such as Box-S, W-box, and WUN-motif, indicating a potential role for *AetCAMTA* genes in various abiotic and biotic stress responses.

In addition, light-responsive motifs including G-box, GATA-motif, and ATC-motif were widely distributed within promoter regions, with G-box motifs occurring nine times across the analyzed promoters.

Core promoter elements, including CAAT-box and TATA-box motifs, were highly represented across all *AetCAMTA* promoters. Notably, CAAT-box elements constituted a large proportion of the identified promoter-associated regulatory elements, suggesting an important role in transcriptional regulation and promoter activity.

### 2.9. Gene Ontology Analysis

Gene Ontology (GO) annotation of AetCAMTA proteins indicated a functional profile consistent with transcriptional regulators. Across the dataset, the predominant Biological Process (BP) terms were associated with regulation of transcription by RNA polymerase II (GO:0006357) and regulation of DNA-templated transcription (GO:0006355). Additional annotations included response to cold (GO:0009409) and positive regulation of DNA-templated transcription (GO:0045893) in several members. In the Molecular Function (MF) category, all AetCAMTA proteins were annotated with DNA binding (GO:0003677) and double-stranded DNA binding (GO:0003690), supporting their role as transcription-associated regulatory proteins. Transcription coregulator activity (GO:0003712) was also detected among several members. Notably, *AetCAMTA5* was additionally annotated with calmodulin-binding activity (GO:0005516), suggesting potential involvement in calcium-mediated signaling pathways. Cellular Component (CC) analysis consistently predicted nuclear localization (GO:0005634) for all AetCAMTA proteins, consistent with their regulatory function in transcription (Table 5). 

### 2.10. Prediction of miRNA Targeting AetCAMTA Genes

**Figure 9:**
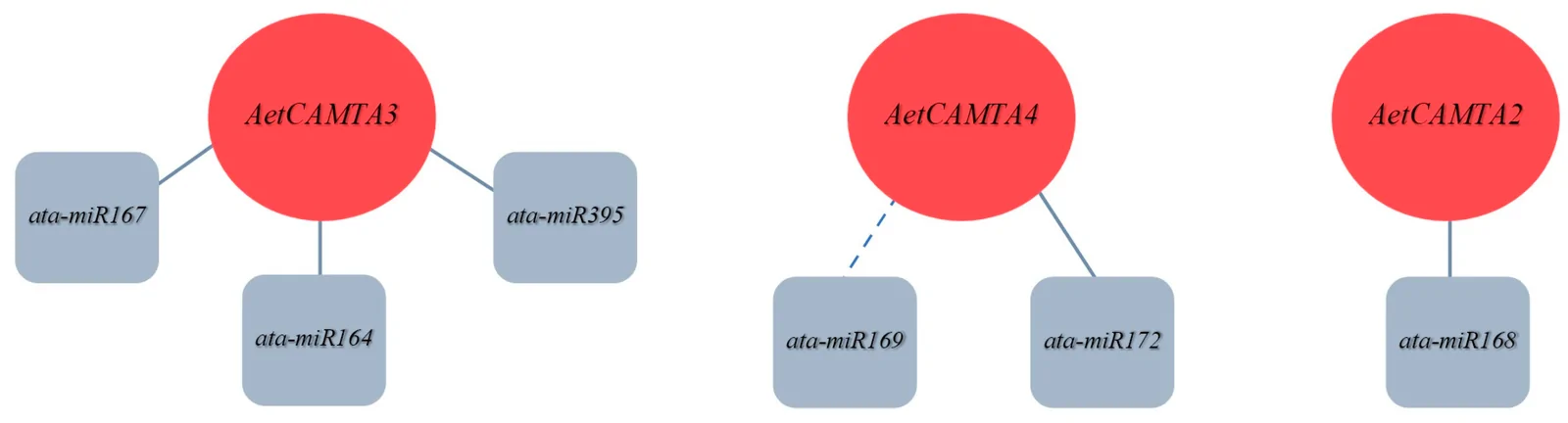
Predicted miRNA–*AetCAMTA* interaction network in *Ae. tauschii*. The network illustrates predicted interactions between *A. tauschii* miRNAs and *AetCAMTA* genes generated using psRNATarget and visualized in Cytoscape. Rectangular nodes represent miRNAs and circular nodes indicate target genes. Solid lines denote cleavage-mediated inhibition, whereas dashed lines indicate translational repression. Differences in node connectivity reflect variation in predicted post-transcriptional regulation among *AetCAMTA* family members.

Putative miRNA–target interactions between *Ae. tauschii* miRNAs and *AetCAMTA* genes were predicted using the psRNATarget server. A total of 175 mature *Ae. tauschii* miRNAs were screened against the full-length coding DNA sequences (CDS) of *AetCAMTA* genes. The analysis identified several potential miRNA–gene interactions, with *AetCAMTA2*, *AetCAMTA3*, and *AetCAMTA4* predicted as targets of multiple miRNAs, suggesting possible post-transcriptional regulation. Among these genes, *AetCAMTA3* showed the highest number of predicted interactions, followed by *AetCAMTA4*, whereas *AetCAMTA2* exhibited comparatively fewer connections. Specifically, *AetCAMTA3* was predicted to be targeted by *ata-miR164b-3p*, *ata-miR167f-3p*, and members of the *ata-miR395* family. *AetCAMTA4* was predicted to interact with *ata-miR169h-3p* and *ata-miR172a/b-3p*, whereas *AetCAMTA2* contained two predicted binding sites for *ata-miR5168-5p* (Table 6). Most predicted interactions were associated with cleavage-mediated inhibition, while translational repression was predicted only for the interaction between *ata-miR169h-3p* and *AetCAMTA4*. Expectation scores ranged from 4.0 to 4.5, and UPE values indicated accessible target regions for miRNA binding. The regulatory interaction network further highlighted differential connectivity among *AetCAMTA* family members (Figure 9). *AetCAMTA3* exhibited the highest number of predicted miRNA interactions, followed by *AetCAMTA4*, whereas *AetCAMTA2* displayed comparatively lower connectivity. Detailed information regarding predicted miRNA–target interactions, including expectation scores, inhibition types, and UPE values, is provided in Table 6.

### 2.11. Expression Analysis of CAMTA Genes

**Figure 10:**
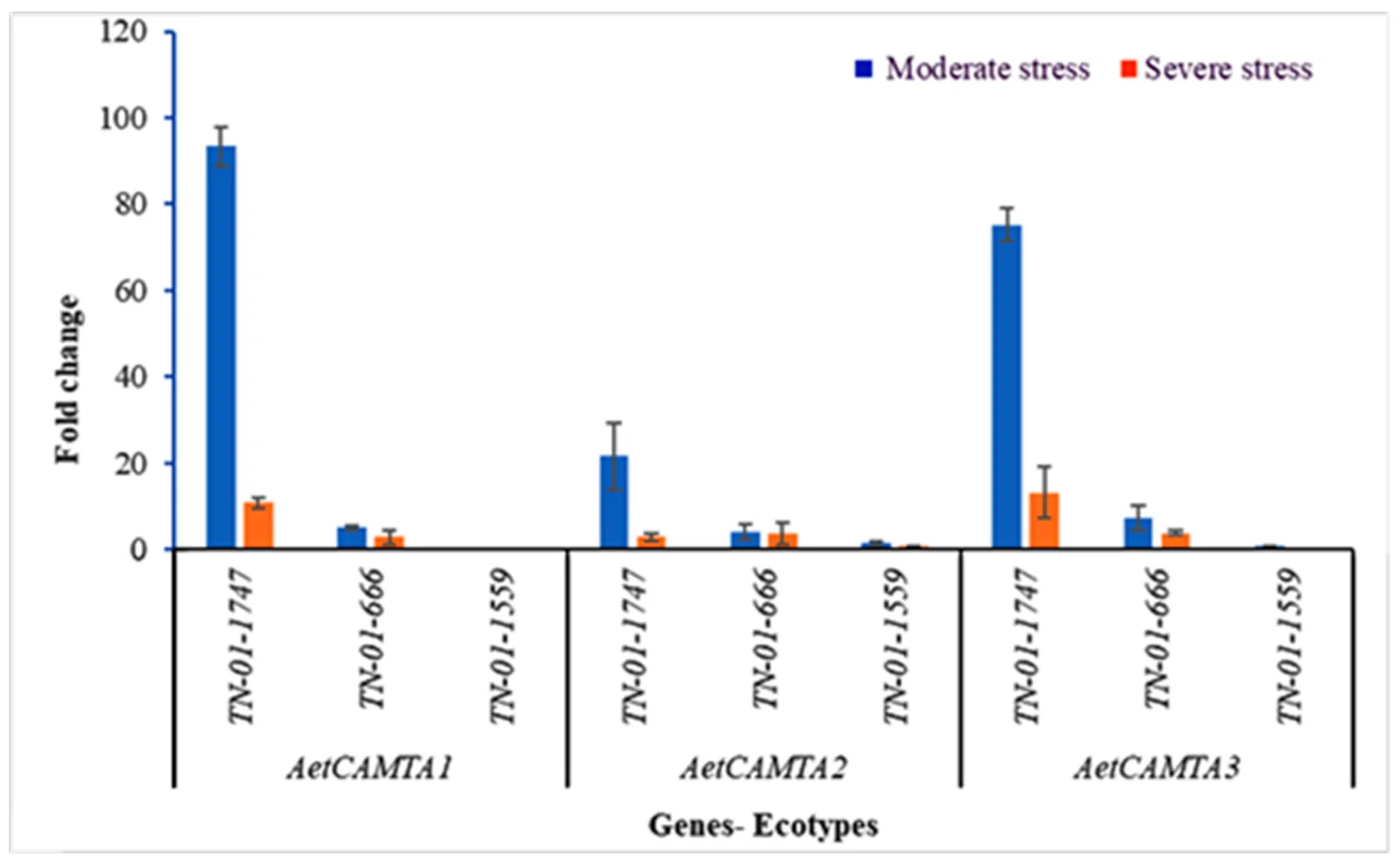
qRT-PCR analysis of the expression of selected *CAMTA* genes including *AetCAMTA1*, *AetCAMTA2*, and *AetCAMTA3* under various drought stress in *Ae. tauschii* ecotypes: Kerman, TN-01-1559 (drought-sensitive), East Azerbaijan, TN-01-666 (moderately tolerant), and Semnan, TN-01-1747 (drought-tolerant), as classified based on the stress tolerance index (STI). Relative expression levels were measured using qRT-PCR with the *18S rRNA* gene used as the internal control. Error bars represent the standard error of the mean (SE) based on two biological replicates. Because of the limited number of biological replicates, no formal statistical significance comparisons were performed, and the results are presented descriptively.

Based on the STI analysis of the 15 *Ae. tauschii* ecotypes, three contrasting ecotypes were selected for Quantitative real-time PCR (qRT-PCR) analysis, including the drought-tolerant ecotype TN-01-1747 from Semnan, the moderately tolerant ecotype TN-01-666 from East Azerbaijan, and the drought-sensitive ecotype TN-01-1559 from Kerman. To provide an initial experimental assessment of drought-responsive transcription within the *AetCAMTA* gene family, the transcriptional responses of three genes (*AetCAMTA1*, *AetCAMTA2*, and *AetCAMTA3*) to moderate and severe drought stress were investigated in leaf tissues of three *Ae. tauschii* ecotypes 24 h after drought induction, using qRT-PCR. Analysis of the melting curves showed that all primer pairs produced a single dominant peak, confirming amplification specificity and the reliability of the qRT-PCR assays. In addition, agarose gel electrophoresis of the PCR products on 2% gels revealed single bands of the expected sizes for all primers, further validating their specificity. Distinct gene-expression patterns were observed among ecotypes and across drought-stress intensities (Figure 10). *AetCAMTA1* showed the highest induction in TN-01-1747 under moderate drought stress (93.48-fold), followed by a marked decline under severe stress (10.79-fold). A similar trend was observed in TN-01-666, whereas TN-01-1559 exhibited only minor changes in expression. *AetCAMTA2* reached its maximum expression in TN-01-1747 under moderate stress (21.66-fold) and then decreased sharply under severe stress to 2.83-fold. TN-01-666 showed a slight reduction, while TN-01-1559, which initially had low expression, increased marginally under severe stress. *AetCAMTA3* was also strongly induced under moderate drought in TN-01-1747 (75.28-fold) and TN-01-666 (7.48-fold), but its expression declined under severe stress. In TN-01-1559, *AetCAMTA3* showed only a slight decrease under both stress levels. Overall, the expression profiles suggested that transcript abundance of all three genes was highest under moderate drought stress, particularly in TN-01-1747, whereas severe drought was generally associated with lower expression. These results indicate that *CAMTA* gene expression is both stress-intensity-dependent and genotype-specific. 

## 3. Discussion

By integrating phenotypic evaluation of drought responses with genome-wide identification, evolutionary and structural characterization, regulatory element profiling, and expression analysis, the present study places the *Ae. tauschii* CAMTA gene family within a broader framework of drought-adaptive regulatory networks in a key wild progenitor of bread wheat. Rather than treating each analytical layer as an isolated descriptive outcome, the discussion below focuses on what these combined layers indicate about the biological role, evolutionary constraints, and regulatory logic of *AetCAMTA* genes, while explicitly distinguishing associative from causal evidence. 

Although Yang et al. [15] identified five *AetCAMTA* members as part of their genome-wide characterization of the wheat CAMTA family, and Wang et al. [57] functionally validated *TaCAMTA1b-B.1*, the present study extends these earlier findings by focusing specifically on *Ae. tauschii*. We integrated comprehensive in silico analyses, including gene structure, *cis*-regulatory element, and miRNA-target predictions, with physiological drought-tolerance screening of 15 *Ae. tauschii* ecotypes. Furthermore, expression profiling of *AetCAMTA* genes in contrasting drought-tolerant and drought-sensitive ecotypes under progressive drought provides novel associative evidence linking transcriptional regulation with variation in drought tolerance in *Ae. tauschii*. Nevertheless, these findings remain correlative, and definitive functional characterization will require further validation, for example, through transgenic or gene-editing approaches.

The pronounced genotype × stress interaction and the divergent PDW profiles among ecotypes indicate the existence of discrete physiological coping mechanisms for water deficit, rather than a synchronized stress response. Notably, the robust performance of TN-01-1747 (Semnan) across both PDW and STI indices suggests a superior ability to sustain growth during water limitation compared to TN-01-1559 (Kerman), consistent with the principle that drought-induced yield loss is driven by impaired carbon fixation and biomass partitioning [58,59,60,61,62,63,64]. Crucially, the enhanced discriminatory power of STI over TOL and SSI reflects a biological reality: by integrating both stress and non-stress productivity, STI’s high concordance with GMP, MP, and HARM underscores that drought tolerance in these *Ae. tauschii* ecotypes is fundamentally linked to biomass stability, allowing for consistent productivity regardless of water availability [54,56,65,66,67]. The phenotypic stratification of ecotypes into sensitive, semi-tolerant, and tolerant groups served as the primary rationale—rather than an ex post facto observation—for investigating *CAMTA* genes as candidate contributors to this differential stability. 

The observed consistency in intron number, the CG-1/ANK/IQ domain architecture, and predicted secondary/tertiary folds should be interpreted not merely as a catalog of structural features, but as evidence of the evolutionary conservation of calcium-decoding functions in CAMTA proteins across the Poaceae. Given that CG-1 mediates sequence-specific DNA binding, ANKs facilitate protein–protein assembly, and IQ motifs confer calmodulin-dependent regulation, the co-preservation of these three domains across all *AetCAMTA* paralogs suggests that the capacity to couple calcium signaling with transcriptional regulation has been strongly conserved throughout evolution [12,16,26,27,68,69,70]. Furthermore, the relatively high proportion of coil regions (49–60%) alongside conserved α-helical content may provide the conformational flexibility necessary for interactions with regulatory partners, a finding consistent with the established link between intrinsic protein flexibility and signal-responsive transcriptional regulation [69,71]. Taken together with the phylogenetic clustering of AetCAMTA proteins with their TaCAMTA orthologs, this structural conservation implies the retention of ancestral CAMTA regulatory features within the D genome of hexaploid wheat rather than extensive functional divergence following polyploidization. However, the distinct phylogenetic placement of *AetCAMTA5* suggests a potential for functional specialization or divergence, a hypothesis that necessitates experimental validation rather than further comparative analysis [72,73,74,75]. 

The concurrent presence of diverse *cis*-regulatory elements—including ABRE, MBS, and various light- and hormone-responsive motifs—within *AetCAMTA* promoters, combined with predicted targeting by multiple stress- and development-associated miRNA families (e.g., miR164, miR167, miR169, miR172, miR395, and the dual-site interaction of *ata-miR5168-5p* with *AetCAMTA2*), challenges the notion of a simple, linear regulatory model for CAMTA expression. Instead, this combinatorial regulatory landscape suggests that *AetCAMTA* genes function as convergence points for multiple signaling inputs, including ABA- and Ca^2+^-associated pathways, to coordinate complex transcriptional responses [76,77,78,79,80]. The differential miRNA targeting profiles observed across paralogs—with *AetCAMTA3* and *AetCAMTA4* harboring a higher density of predicted binding sites than *AetCAMTA2*—further suggest that post-transcriptional fine-tuning is not uniformly distributed across the family. This implies that individual members may be modulated with distinct temporal or spatial sensitivities, a pattern consistent with the layered transcriptional/post-transcriptional control documented in other plant stress-responsive gene families [81,82,83,84,85,86,87]. While this regulatory complexity provides a compelling hypothesis for the differential expression of *AetCAMTAs* under drought, these predicted interactions do not, by themselves, establish functional regulation or demonstrate a contribution to the tolerant phenotype. Consequently, experimental validation is essential to elucidate the functional significance of these putative miRNA–target interactions.

Our qRT-PCR results suggest that the drought-tolerant ecotype TN-01-1747 exhibits a more robust induction of *AetCAMTA1*, *AetCAMTA2*, and *AetCAMTA3* under water deficit compared to the sensitive ecotype TN-01-1559. Notably, these expression patterns were modulated by stress intensity; for instance, *AetCAMTA1* showed peak induction under moderate drought, which declined under severe stress. This biphasic response may indicate that CAMTA-associated signaling is particularly critical during the acclimation phase, whereas severe stress likely imposes broader metabolic and transcriptional constraints that override specific signaling pathways [88]. While intriguing, this interpretation remains speculative in the absence of time-course experiments and functional validation. These patterns support the hypothesis that *AetCAMTA* activation contributes to the physiological stability observed in TN-01-1747. The trajectory of *AetCAMTA1* is compatible with a role in early acclimation signaling, a pattern similarly reported for other plant CAMTA homologs under progressive water deficit [1,15,89]. However, this expression pattern is inherently correlative: elevated transcript abundance in the tolerant ecotype could be a downstream consequence of a superior water status or antioxidant capacity rather than a primary driver of tolerance. Thus, the genotype-dependent differences we observed are best interpreted as one component of a coherent molecular signature that co-occurs with superior drought performance, alongside the promoter architecture, miRNA regulation, and structural conservation described previously, rather than as definitive evidence of a causal regulatory role. Comparable expression–phenotype correlations have been documented in *Arabidopsis* and *Solanum lycopersicum*, where CAMTA-related genes are implicated in ABA signaling and stomatal regulation. In the latter, the knockdown of the tomato *SlSR1L* member directly reduced drought tolerance, accelerated water loss, and restricted root biomass [28,29,90]. Such studies provide a valuable template for future loss-of-function investigations required to transition from association to functional mechanism in *Ae. tauschii*. Finally, it should be noted that the suitability of 18S rRNA as a reference gene was not experimentally validated in the present study. As 18S rRNA is ribosomal rather than messenger RNA, its stability may fluctuate differently from mRNA targets under varying stress conditions. Future research should prioritize the validation of a robust panel of candidate reference genes (e.g., *actin* and *elongation factor 1-alpha*) using algorithms such as geNorm, NormFinder, or BestKeeper to ensure the normalization accuracy of transcriptional data across these ecotypes and water regimes. 

In all, our integrated findings—spanning evolutionary conservation, combinatorial regulatory architecture, and contrasting expression profiles—establish a robust molecular association between the *AetCAMTA* family and drought adaptation in *Ae. tauschii*. However, transitioning from correlative insight to causal mechanism mandates rigorous functional interrogation using CRISPR/Cas editing or VIGS (virus-induced gene silencing) systems. Validating *AetCAMTA1* and *AetCAMTA3* through these direct phenotypic assays represents an essential milestone before their translational application in breeding climate-resilient cereal crops.

## 4. Materials and Methods

### 4.1. Plant Material

Fifteen *Ae. tauschii* ecotypes obtained from the National Plant Gene Bank of Iran (Karaj, Iran) were used in this study. Information regarding the origin of the ecotypes is presented in Table 7. Seeds were vernalized at 2–4 °C for 30 days prior to sowing to ensure uniform germination and seedling establishment. The experiment was conducted in a greenhouse at the University of Mohaghegh Ardabili (Ardabil, Iran) as a factorial arrangement based on a completely randomized design with three replications. Plants were grown in 6 kg plastic pots (30 cm diameter × 30 cm height), and the physicochemical properties of the soil are presented in Table 8. After germination, three seedlings were maintained per pot. Three independent pots were used for each drought treatment per ecotype, resulting in three biological replicates per treatment and a total of 135 pots for the 15 ecotypes. No supplementary fertilization was applied during the experimental period. Greenhouse conditions were maintained at 20 ± 3 °C during the light period and 16 ± 3 °C during the dark period with 40% relative humidity and a 16 h light/8 h dark photoperiod. Drought treatments were applied 30 days after planting at three soil moisture levels corresponding to 100% (control), 60%, and 30% field capacity (FC) according to Jacob and Clark [91]. To maintain the desired soil moisture levels, pots were weighed using a digital scale and the required amount of water was added approximately every four days. Sixty days after the initiation of drought stress, the aboveground plant parts were harvested to determine PDW. Samples were dried in a laboratory digital oven (Behsan Co. Tehran, Iran) at 70 °C for 72 h until a constant weight was reached, and then weighed using a digital balance with an accuracy of ±0.001 g.

### 4.2. Drought Tolerance Indices

The ecotypes’ tolerance and sensitivity to drought stress were assessed by comparing their performance (PDW) under non-stress conditions (Yp) and stress conditions (Ys) according to the formulas presented in Table 9. Based on STI values, three contrasting *Ae. tauschii* ecotypes were selected for expression profiling of *AetCAMTA* genes under drought stress conditions. The indices were calculated separately for moderate (60% FC) and severe drought stress (30% FC) treatments.

### 4.3. Statistical Analysis

Analysis of variance (ANOVA) and mean comparisons were performed using Tukey’s HSD test at *p* < 0.01 in the STAR (Statistical Tool for Agricultural Research) softwar version 2.0.1 [96]. Drought tolerance indices were calculated using Microsoft Excel. Heatmap visualization and hierarchical clustering analysis were performed using CIMMiner [97]. based on Euclidean distance and Ward’s clustering method.

### 4.4. RNA Purification and RT-qPCR Analysis

Based on the STI analysis of the 15 *Ae. tauschii* ecotypes, three contrasting ecotypes were selected for qRT-PCR analysis: the drought-tolerant ecotype TN-01-1747 (Semnan), the moderately tolerant ecotype TN-01-666 (East Azerbaijan), and the drought-sensitive ecotype TN-01-1559 (Kerman). To provide an initial experimental assessment of drought-responsive transcription within the *AetCAMTA* gene family, the transcriptional responses of three genes (*AetCAMTA1*, *AetCAMTA2*, and *AetCAMTA3*) to moderate and severe drought stress were investigated by qRT-PCR in leaf tissues collected 24 h after drought induction.

Total RNA was extracted from leaf tissues using the RNX-Plus RNA extraction kit (Sinaclon, Tehran, Iran) according to the manufacturer’s instructions. The concentration and purity of RNA samples were determined using a NanoDrop OneC spectrophotometer (Thermo Scientific, Waltham, MA, USA). RNA integrity was further verified by agarose gel electrophoresis. RNA purity was evaluated based on the absorbance ratios at A260/A280 and A260/A230. First-strand cDNA was synthesized using a cDNA synthesis kit (Yektatajhiz, Tehran, Iran) following the manufacturer’s protocol. Briefly, 1–2 μL of total RNA was reverse-transcribed in a 20 μL reaction mixture containing oligo(dT) primers, dNTPs, RNase inhibitor, reverse transcriptase (M-MLV), and reaction buffer. The reverse transcription reaction was carried out at 42 °C for 60 min, followed by enzyme inactivation at 70 °C for 5 min. The synthesized cDNA samples were stored at −70 °C until further use. 

RT-qPCR was performed using Super SYBR Green qPCR Master Mix (Yektatajhiz, Tehran, Iran). Each 10 μL PCR reaction contained 1 μL of diluted cDNA template, 5 μL of SYBR Green Master Mix, 0.5 μL of each forward and reverse primer, and 3 μL of nuclease-free water. Amplification reactions was performed as described in Table 10, were carried out using a Rotor-Gene Q real-time PCR system (Qiagen, Germany). Technical replicates were averaged for each biological replicate prior to calculation of relative expression, and were not treated as independent observations in accordance with MIQE recommendations [98,99]. Each experiment included two biological replicates with two technical replicates. Among the five identified *AetCAMTA* genes, *AetCAMTA1*, *AetCAMTA2*, and *AetCAMTA3* were selected for preliminary expression analysis to provide an initial experimental assessment of drought-responsive transcription within the gene family.

Relative gene expression levels were calculated using the 2^−ΔΔCt^ method [100]. 18S rRNA was used as the internal reference gene based on its prior application in gene-expression studies in wheat and related *Triticeae* species. However, we acknowledge that reference-gene stability is condition-dependent, and experimental validation of multiple candidate reference genes is recommended for each system [101,102]. All reactions were performed under identical conditions and with the same cDNA input, and no template controls (NTC) were included. 

Gene-specific primers were designed using the Primer3Plus web tool (https://www.bioinformatics.nl/cgi-bin/primer3plus/primer3plus.cgi) (accessed on 26 August 2026) [103] based on the mRNA sequences obtained from the NCBI database. Primer specificity was further verified using NCBI Primer-BLAST (https://www.ncbi.nlm.nih.gov/tools/primer-blast/, accessed on 26 August 2026) [104], and primer properties were evaluated using OligoAnalyzer (version 1.0.2). The designed primers amplified PCR products ranging from 150 to 200 bp (Table 11).

### 4.5. Identification of AetCAMTA Genes in the Ae. tauschii Genome

*CAMTA* genes in the *Ae. tauschii* genome were identified using known CAMTA protein sequences from *Arabidopsis thaliana*, *Medicago truncatula*, *Brassica napus*, and *O. sativa japonica* retrieved from the NCBI and Plant Transcription Factor Database (PlantTFDB). These sequences were used as queries to perform tBLASTn searches against the *Ae. tauschii* genome in the NCBI database. All candidate sequences were further verified for the presence of conserved CAMTA domains (CG-1 DNA-binding domain, ANK, and IQ motifs) using the SMART [105] and Pfam [106] databases (accessed on 26 August 2026). Sequences lacking these conserved domains were excluded. The identified genes were designated with the prefix Aet (for *Ae. tauschii*) followed by CAMTA and a number according to their homology with rice *CAMTA* proteins. Detailed information including protein ID, gene ID, chromosomal location, protein length, molecular weight, isoelectric point (pI), exon number, instability index (II), extinction coefficient, and grand average of hydropathicity (GRAVY) was calculated using the ProtParam tool (https://web.expasy.org/protparam/) (accessed on 26 August 2026) [107]. Subcellular localization was predicted using the Plant-mPLoc server (http://www.csbio.sjtu.edu.cn/bioinf/plant-multi/) (accessed on 26 August 2026) [108]. To investigate evolutionary relationships, a phylogenetic tree was constructed using MEGA-X software (version 10.1.8) [109] using the Maximum Likelihood (ML) method with 1000 bootstrap replicates [110]. The analysis included full-length CAMTA protein sequences from *Ae. tauschii*, *Triticum aestivum*, and *O. sativa japonica*. Evolutionary distances were calculated using the Poisson correction method.

### 4.6. Gene Structure Analysis

The exon–intron organization of *AetCAMTA* genes was analyzed using the Gene Structure Display Server (GSDS 2.0; http://gsds.gao-lab.org/) (accessed on 26 August 2026) [111] by aligning coding sequences with their corresponding genomic sequences.

### 4.7. Conserved Motif and Domain Analysis

Conserved motifs in CAMTA protein sequences were identified using the MEME Suite (https://meme-suite.org/) [112] with default parameters. The maximum number of motifs was set to 10, and motif widths ranged from 6 to 50 amino acids. Motif annotations were validated using InterProScan (https://www.ebi.ac.uk/interpro/) (accessed on 26 August 2026) [113], and only consistent results were retained. The distribution of motifs was visualized using DOGS (V 2.0) (Domain Graphical Structure; http://dog.biocuckoo.org/) (accessed on 26 August 2026) [114]. Conserved domains including CG-1, ANK, and IQ motifs were identified using the SMART [105] database (http://smart.embl-heidelberg.de/) (accessed on 26 August 2026).

### 4.8. Secondary Structure Prediction

Secondary structures of AetCAMTA proteins were predicted using PSIPRED v4.0 (http://bioinf.cs.ucl.ac.uk/psipred/) (accessed on 26 August 2026) [115] with default parameters. Residues were categorized into α-helix, β-strand, or coil conformations based on position-specific scoring matrices generated by PSI-BLAST [116].

### 4.9. Protein 3D Structure Modeling, Refinement, and Validation

Three-dimensional structures of AetCAMTA proteins were predicted using the SWISS-MODEL server (https://swissmodel.expasy.org/) (accessed on 26 August 2026) [117] based on homology modeling. The best models were selected based on the highest Global Model Quality Estimation (GMQE) score and sequence identity. Selected models were refined using GalaxyRefine (accessed on 26 August 2026) [118] to improve local structural quality. Further energy minimization was performed using UCSF Chimera (V 1.16) [119] to resolve steric clashes. Structural quality was assessed using the MolProbity server (http://molprobity.biochem.duke.edu/) (accessed on 26 August 2026) [120], including MolProbity score, clashscore, and Ramachandran plot statistics. Models with >95% residues in favored regions and <1.5% in outlier regions were considered high quality. Final structures were visualized using PyMOL (V 2.5.5). [121] Conserved domains identified via SMART [105] were mapped onto 3D structures and color-coded for structural interpretation.

### 4.10. Promoter Cis-Acting Element Analysis

Promoter regions (1500 bp upstream of the ATG start codon) of *AetCAMTA* genes were retrieved and analyzed for cis-acting regulatory elements using the PlantCARE database https://bioinformatics.psb.ugent.be/webtools/plantcare/html/ (accessed on 26 August 2026) [122]. A 1500 bp upstream region was selected as it typically contains the majority of cis-regulatory elements in plant promoters. Stress- and hormone-responsive elements were identified.

### 4.11. GO Annotation

Functional annotation of AetCAMTA proteins was performed using GO terms retrieved from UniProtKB (accessed on 26 August 2026) [123] and NCBI databases. GO terms were categorized into Biological Process (BP), Molecular Function (MF), and Cellular Component (CC). Electronically inferred annotations (IEA) were included for functional interpretation [124].

### 4.12. miRNA Target Prediction

Potential miRNA–target interactions between *Ae. tauschii* miRNAs and *AetCAMTA* genes were predicted using the psRNATarget online server (https://www.zhaolab.org/psRNATarget/) (accessed on 26 August 2026) [125]. The scoring schema was based on complementarity scoring implemented in the psRNATarget algorithm. A total of 175 mature *A. tauschii* miRNA sequences obtained from miRBase (v22) [126] were used as query sequences, while the full-length coding sequences of *AetCAMTA* genes were used as targets. Predictions were performed using default parameters with a maximum expectation score of 5.0. Target accessibility was evaluated based on the unpaired energy (UPE) required to expose the target site. Predicted miRNA–target interactions were visualized as a regulatory network using Cytoscape software (version 3.10.4) [127].

## 5. Conclusions

This study identified and characterized five *AetCAMTA* genes in *Ae. tauschii* and explored their potential involvement in drought-responsive processes through integrated bioinformatic, phenotypic, and transcriptional analyses. The conserved domain organization, gene structures, and predicted regulatory features of these genes indicated a high degree of evolutionary conservation within the CAMTA family. Phenotypic assessment revealed substantial variation in drought tolerance among the evaluated *Ae. tauschii* ecotypes. Ecotype TN-01-1747 displayed comparatively greater drought tolerance, whereas TN-01-1559 was the most drought-sensitive. Furthermore, transcriptional analysis showed that the expression patterns of *AetCAMTA1*, *AetCAMTA2*, and *AetCAMTA3* varied according to both drought treatment and genotype. Notably, these genes generally exhibited higher transcript accumulation in the drought-tolerant ecotype under stress conditions, providing preliminary evidence of their potential association with drought-responsive mechanisms. Nevertheless, the relatively limited number of analyzed genes and biological replicates warrants cautious interpretation of these results. Future investigations should include a greater number of independent biological replicates, additional CAMTA family members, and robust RT–qPCR normalization based on the geometric mean of two or more validated reference genes. Overall, this study provides a foundation for further investigation of CAMTA-mediated drought responses in *Ae. tauschii* and clarify their roles in drought adaptation and potential utility for improving drought resilience in wheat and related cereal crops.

## Figures and Tables

**Table 1:** Analysis of variance for *Ae. tauschii* morphological and biochemical traits grown under drought stress.

Source of Variation	Drought Stress (T)	Ecotype (G)	T × G	Error	C.V. ^1^ (%)
df	2	14	28	90	-
MS (PDW ^2^)	239.057 **^3^	20.695 **	2.889 **	1.019	14.73

^1^ (C.V.) coefficient of variation, ^2^ (PDW) Plant dry weight. ^3^ (**) significance at 1% probability levels.

**Table 2:** Characteristics of the five CAMTA genes identified in *Ae. tauschii*.

Gene Name	Protein ID	Gene ID ^4^	Chromosome Location	Length (aa ^5^)	Mw ^6^ (Da)	PI ^7^	Exon No	Instability Index (II)	Extinction Coefficients	GRAVY	Subcellular Localization
*AetCAMTA1*	XP_020189402.1	109775063	3D .NC_053037.2 (548840856..548847055, complement)	832	94,182.35	6.53	11	39.70	121,530	−0.508	Nucleus
*AetCAMTA2*	XP_020179695.1	109765312	4D .NC_053038.2 (478766355..478775059, complement)	1066	119,164.08	5.66	13	51.52	148,545	−0.552	Nucleus
*AetCAMTA3*	XP_020196708.1	109782494	2D .NC_053036.2 (114975938..114984445)	1026	113,914.58	5.59	13	44.14	122,575	−0.515	Nucleus
*AetCAMTA4*	XP_020147564.1	109732785	2D .NC_053036.2 (354027111..354035539)	1022	114,106.03	5.32	12	49.89	135,650	−0.517	Nucleus
*AetCAMTA5*	XP_020186933.1	109772636	2D .NC_053036.2 (239760134..239774689, complement	891	100,279.42	9.00	13	39.38	124,845	−0.515	Nucleus

^4^ (ID) identity, ^5^ (aa) amino acids, ^6^ (MW) molecular weight, ^7^ (pI) isoelectric point.

**Table 3:** Protein secondary structure composition of CAMTA genes in *Ae. tauschii*.

Second Structure Type	*AetCAMTA1*	*AetCAMTA2*	*AetCAMTA2*	*AetCAMTA4*	*AetCAMTA5*
Alpha-Helix	41.22%	35.64%	34.21%	36.59%	42.98%
Coil (Loop)	49.63%	59.75%	56.92%	58.12%	50.05%
Beta-Sheet	9.13%	4.59%	8.86%	5.28%	6.95%

Structural validation analyses supported the reliability of the predicted models. MolProbity scores ranged from 0.72 to 0.81, and low steric clash scores were observed across the generated structures. Ramachandran plot analysis further demonstrated that 95.60–96.62% of residues were located within favored regions, whereas outlier residues remained below 1.1%, supporting the stereochemical quality of the predicted models. Detailed structural validation parameters are summarized in Supplementary Table S3.

**Table 4:** Analysis of cis-elements in the promoter regions (1500 bp upstream region from the translation start site) of *AetCAMTA* genes.

Cis-Elements	*AetCAMTA1*	*AetCAMTA2*	*AetCAMTA3*	*AetCAMTA4*	*AetCAMTA5*
**A-box**	2	2	1	2	1
**ABRE**	6	5	3	7	2
**ABRE3a**	1	0	0	3	0
**ABRE4**	1	0	0	3	0
**ARE**	0	0	1	0	1
**ATC-motif**	0	0	1	0	0
**AuxRR-core**	0	1	1	0	0
**CAAT-box**	7	15	10	5	38
**CGTCA-motif**	5	3	5	3	2
**G-Box**	3	3	1	1	1
**GARE-motif**	0	1	0	0	0
**GATA-motif**	0	0	0	0	1
**GC-motif**	2	2	4	0	0
**GCN4_motif**	0	1	0	0	0
**GT1-motif**	0	1	0	0	2
**I-box**	0	0	1	0	0
**LTR**	1	0	1	1	0
**LAMP-element**	0	0	1	0	0
**MBS**	0	1	0	0	1
**MRE**	0	0	0	0	1
**MYB**	1	2	4	2	2
**MYC**	2	1	3	2	3
**Myb**	0	1	0	0	1
**P-box**	0	0	1	0	1
**Sp1**	1	1	2	1	2
**TATA-box**	1	13	16	3	25
**TC-rich repeats**	0	0	1	0	1
**TCCC-motif**	2	0	1	0	0
**TCT-motif**	0	0	0	0	1
**TGA-element**	0	1	1	0	0
**TGACG-motif**	5	3	5	3	2
**WUN-motif**	1	0	0	0	0
**WRE3**	2	2	2	3	2
**box S**	0	1	0	0	0
**chs-Unit 1 m1**	0	1	1	0	0

**Table 5:** GO annotation of AetCAMTA proteins.

Protein Names	UniProt ID	Biological Process IDs	Biological Process Molecular Names	Molecular Function IDs	Molecular Function Names	Cellular Component IDs	Cellular Components	Evidence
*AetCAMTA1*	A0A453GCS2	(BP ^8^):GO:0006357	transcription regulation by RNA polymerase II	(MF ^9^):GO:0003677	DNA binding	(CC ^10^):GO:0005634	nucleus	IEA ^11^
				(MF):GO:0003690	double-stranded DNA binding			
				(MF):GO:0003712	transcription coregulator activity			
*AetCAMTA2*	A0A453IZH6	(BP):GO:0006355	regulation of DNA-templated transcription	(MF):GO:0003677	DNA binding	(CC):GO:0005634	nucleus	IEA
		(BP):GO:0006357	regulation of transcription by RNA polymerase II	(MF):GO:0003690	double-stranded DNA binding			
		(BP):GO:0009409	response to cold	(MF):GO:0003712	transcription coregulator activity			
*AetCAMTA2*	A0A453B211	(BP):GO:0006357	regulation of transcription by RNA polymerase II	(MF):GO:0003677	DNA binding	(CC):GO:0005634	nucleus	IEA
		(BP):GO:0009409	response to cold	(MF):GO:0003690	double-stranded DNA binding			
		(BP):GO:0045893	positive regulation of DNA-templated transcription	(MF):GO:0003712	transcription coregulator activity			
*AetCAMTA4*	A0A453BV81	(BP):GO:0006355	regulation of DNA-templated transcription	(MF):GO:0003677	DNA binding	(CC):GO:0005634	nucleus	IEA
		(BP):GO:0006357	regulation of transcription by RNA polymerase II	(MF):GO:0003690	double-stranded DNA binding			
		(BP):GO:0009409	response to cold	(MF):GO:0003712	transcription coregulator activity			
*AetCAMTA5*	A0A453BHL8	(BP):GO:0006355	regulation of DNA-templated transcription	(MF):GO:0003677	DNA binding	(CC):GO:0005634	nucleus	IEA
		(BP):GO:0006357	regulation of transcription by RNA polymerase II	(MF):GO:0003690	double-stranded DNA binding			
		(BP):GO:0045893	positive regulation of DNA-templated transcription	(MF):GO:0003712	transcription coregulator activity			
				(MF):GO:0005516	calmodulin binding			

(BP) ^8^ Biological Process, (MF) ^9^ Molecular Function, (CC) ^10^ Cellular Component, (IEA) ^11^ Inferred from Electronic Annotation.

**Table 6:** Potential miRNA targets of *CAMTA* genes in *Ae. tauschii*.

miRNA ID.	Target Gene	Expectation	UPE	Alignment	Inhibition	Multiplicity
*ata-miR164b-3p*	*AetCAMTA3*	4	−1	miRNA: AUGUGCCUUUCUUCUCCACC	Cleavage	1
				Target: GGUGGAGGAGAAAGGGACGGG		
*ata-miR167f-3p*	*AetCAMTA3*	4.5	−1	miRNA:CAGAUCAUGCUGCAGCUUCAU	Cleavage	1
				Target: AUGAACUUGCAGC-UGAUCUG		
*ata-miR169h-3p*	*AetCAMTA4*	4.5	−1	miRNA: GCAAGUUGUUCUUGGCUAGC	Translation	1
				Target: CCUAGACAGCAGUAAUUUGC		
*ata-miR172 a/b-3p*	*AetCAMTA4*	4.5	−1	miRNA: AGAAUCUUGAUGAUGCUGCGU	Cleavage	1
				Target: UUGCAGCUGCAUCAAGGUUAU		
*ata-miR395a/c/d/e/f 3p*	*AetCAMTA3*	4.5	−1	miRNA: UGAAGUGUUUGGGGGAACUC	Cleavage	1
				Target: GAUAUCCCCCAGAUACUGCA		
*ata-miR5168-5p*	*AetCAMTA2*	4.5	−1	miRNA: GGGUUGUUGUCUGGUUCAAGG	Cleavage	2
				Target: AUUUGAACAAGAAGAUAACCU		
*ata-miR5168-5p*	*AetCAMTA2*	4.5	−1	miRNA: GGGUUGUUGUCUGGUUCAAGG	Cleavage	2
				Target: AGUUGCAACAGACAGCAGCAC		

**Table 7:** Information on the origin of *Ae. tauschii* ecotypes used in this study.

Number	Gene Bank Code	Province
1	TN-01-304	West Azerbaijan
2	TN-01-563	Rasht
3	TN-01-666	East Azerbaijan
4	TN-01-707	Gorgan
5	TN-01-804	Markazi
6	TN-01-1011	Hormozgan
7	TN-01-1336	Kurdistan
8	TN-01-1559	Kerman
9	TN-01-1695	Tehran
10	TN-01-1747	Semnan
11	TN-01-1770	Mazandaran 1
12	TN-01-1773	Mazandaran 2
13	TN-01-2015	Nur
14	TN-01-2018	Ramsar
15	TN-01-2121	Khorasan

**Table 8:** Physicochemical characteristics of the soil used in experimental pots.

Characteristic	pH	Texture	Electrical Conductivity (ds m^−1^)	Silt (%)	Clay (%)	Sand (%)	Organic Carbon (%)	Nitrogen (%)	Zinc (mg kg^−1^)	Phosphorus (mg kg^−1^)	Potassium (mg kg^−1^)
Values	7.4	Loam	0.97	42	19.5	38.5	0.72	0.07	1.12	28.3	275

**Table 9:** Different drought tolerance indices used in this research with their calculation formulas.

Abbreviation	Drought Tolerance Index	Formula	Reference
TOL	Tolerance Index	$TOL=Y_{p}-Y_{s}$	[56]
MP	Mean Productivity	$MP=\left(Y_{p}+Y_{s}\right)/2$	[56]
STI	Stress Tolerance Index	$STI=\left(Y_{p}\right)\left(Y_{s}\right)/{\left({\bar{Y}}_{p}\right)}^{2}$	[54]
GMP	Geometric Mean Productivity	$GMP=\sqrt{\left(Y_{p}\times Y_{s}\right)}$	[54]
HM	Harmonic mean	$HM=2\left(Y_{p}\times Y_{s}\right)/\left(Y_{p}+Y_{s}\right)$	[92]
SSI	Stress susceptibility index	$SSI=\left(1-\left(Y_{s}/Y_{p}\right)\right)/SI$	[55]
YI	Yield index	$YI=Y_{s}/{\bar{Y}}_{s}$	[93]
YSI	Yield stability index	$YSI=Y_{s}/Y_{p}$	[94]
YRP	Percentage of yield decrease	$YRP=\frac{\left(Y_{p}-Y_{s}\right)}{Y_{p}}\times 100$	[95]

*Y_p_*, plant dry weight under non-stress (control) conditions; *Y_s_*, plant dry weight under drought stress conditions; *Ȳ_p_* mean plant dry weight of all ecotypes under non-stress conditions; *Ȳ_s_* mean plant dry weight of all ecotypes under stress conditions; and SI (stress intensity) = 1 − (*Ȳ_s_/Ȳ_p_*).

**Table 10:** Thermal cycling conditions used for RT-qPCR analysis.

Step	Temperature	Time	Cycles
Initial denaturation	95 °C	3 min	1
Denaturation	95 °C	10 s	
Annealing	60 °C	20 s	40
Extension	72 °C	20 s	
Melt curve	72–95 °C	5 s per step	1

**Table 11:** Primer sequences used for RT-qPCR analysis.

Genes		Primers (5′→3′)	Product Size (bp)
*AetCAMTA1*	F	ATTATTGGGAACGGGAAACC	173
	R	GACTGGACATTTGGGAGTGG	
*AetCAMTA2*	F	CCACCTTCAAACCCTCAACCT	169
	R	GGCAGCATTCCTCACAGCAC	
*AetCAMTA3*	F	CTATTGGTTCCCTGATGCTTG	183
	R	TGCCCTTCCTTGCCTAACAC	
*18S rRNA*	F	GCATGGGATAACACCACAGG	190
	R	GGCATCGTTTATGGTTGAGAC	

## Data Availability

The original contributions presented in this study are included in the article/Supplementary Material. Further inquiries can be directed to the corresponding author(s).

## Supplementary Materials

The following supporting information can be downloaded at: https://www.mdpi.com/article/10.3390/ijms27177790/s1.

## Abbreviations

The following abbreviations are used in this manuscript:

CAMTA	Calmodulin-Binding Transcription Activator
CaM	Calmodulin
CaMB	Ca^2+^-dependent CaM-binding domain
CG-1	CG-1 DNA-binding domain
ANK	Ankyrin repeat
NLS	Nuclear localization signal
CDS	Coding DNA sequence
UTR	Untranslated region
GO	Gene Ontology
BP	Biological Process
MF	Molecular Function
CC	Cellular Component
miRNA	microRNA
qRT-PCR	Quantitative real-time PCR
PDW	Plant dry weight
FC	Field capacity
DEPC	Diethyl pyrocarbonate water
PEG	Polyethylene glycol
STI	Stress Tolerance Index
SSI	Stress Susceptibility Index
TOL	Tolerance Index
MP	Mean Productivity
GMP	Geometric Mean Productivity
HARM	Harmonic Mean
YSI	Yield Stability Index
YI	Yield Index
YS	Yield under stress
YP	Yield under non-stress conditions
ABRE	ABA-responsive element
MBS	MYB-binding site
AuxRE	Auxin-responsive element
AuxRR	Auxin-responsive element
ABA	Abscisic acid
SA	Salicylic acid
MeJA	Methyl jasmonate
NHP	N-hydroxypipecolic acid
Pip	Pipecolic acid
ALMT1	Aluminum-activated malate transporter 1
GMQE	Global Model Quality Estimation
