# Supplementary material for: Genome-Wide Characterization of Calmodulin-Binding Transcription Activators Genes in Aegilops tauschii
Source: Int J Mol Sci. 2026 Aug 31;27(17):7790. doi: 10.3390/ijms27177790 (PMC13566694; doi:10.3390/ijms27177790)
Supplement: Supplementary file 1 [file ijms-27-07790-s001.zip › ijms-4468578-supplementary.pdf]

Supplementary Table S1. Motif composition, positional coordinates, and conserved domain architecture of AetCAMTA proteins.

Protein	Motif composition	Conserved domains
AetCAMTA1	M1(15–62), M2(63–112), M3(335–359), M4(531–559), M5(710–759)	CG-1, IPT/TIG, ANK, IQ
AetCAMTA2	M1(21–68), M2(69–118), M3(509–533), M4(701–729), M5(896–945)	CG-1, IPT/TIG, ANK, IQ
AetCAMTA3	M1(22–69), M2(70–119), M3(473–497), M4(659–687), M5(854–903)	CG-1, IPT/TIG, ANK, IQ
AetCAMTA4	M1(13–60), M2(61–110), M3(482–506), M4(667–695), M5(867–916)	CG-1, IPT/TIG, ANK, IQ
AetCAMTA5	M1(34–81), M2(82–131), M3(377–401), M4(565–593), M5(753–802)	CG-1, IPT/TIG, ANK, IQ

Supplementary Table S2. Functional annotation and statistical significance (E-values) of conserved motifs identified in AetCAMTA proteins.

Motif	Annotation	E-value
M1	CG-1 associated	6.7×10^{-905}
M2	CG-1 associated	1.6×10^{-80}
M3	IPT/TIG associated	1.7×10^{-49}
M4	Ankyrin repeat associated	6.9×10^{-62}
M5	IQ/calmodulin-binding associated	1.8×10^{-29}

Supplementary Table S3. Structural validation statistics of refined AetCAMTA three-dimensional models generated by SWISS-MODEL- UCSF Chimera and evaluated using MolProbity.

Protein	MolProbity score	Clashscore	Rama favored (%)	Rama outliers (%)
AetCAMTA1	0.74	0.00	96.38	0.85
AetCAMTA2	0.79	0.00	95.77	1.03
AetCAMTA3	0.81	0.00	95.60	0.39
AetCAMTA4	0.75	0.00	96.28	0.40
AetCAMTA5	0.72	0.00	96.62	0.56
